# Maternal hypertension and telomere length are associated with weight for age Z score change from birth to 6 months of age in a predominantly Latinx cohort

**DOI:** 10.1186/s12884-025-08213-8

**Published:** 2025-11-12

**Authors:** Janet M. Wojcicki, Mehr Sahota, Jue Lin, Kimberley Coleman-Phox, Laura Jelliffe-Pawlowski, Larry Rand

**Affiliations:** 1 Division of Pediatric Gastroenterology, Hepatology and Nutrition, Department of Pediatrics, University of California, San Francisco, San Francisco, CA USA; 2 Department of Epidemiology and Biostatistics, University of California, San Francisco, San Francisco, CA USA; 3 Department of Biochemistry and Biophysics, University of California, San Francisco, San Francisco, CA USA; 4 Preterm Birth Initiative, University of California, San Francisco, San Francisco, CA USA; 5 Department of Obstetrics, Gynecology and Reproductive Health Sciences, University of California, San Francisco, San Francisco, CA USA; 6 Department of Pediatrics, 550 16th Street, San Francisco, CA 94134-0136 USA

**Keywords:** Telomere, Telomere length, Rapid infant weight gain, Weight for age Z change

## Abstract

**Background:**

Weight gain in the first six months of life is a predictor of future obesity with rapid infant weight gain (RIWG) (change > 0.67 SD in weight for age Z score from birth to 6 months of age), a strong predictor for obesity at age 5.

**Methods:**

We recruited a multi-racial birth cohort of primarily Latinx mothers and newborns at two San Francisco hospitals. Dried blood spots were taken from infants for leukocyte telomere length (LTL) analysis. Multivariable models were used to evaluate risk factors for RIWG, weight for age Z score (WAZ) change and WAZ at 6 months including gestational and/or pre-existing hypertension and LTL. Separate models were run for term infants ( > = 37 weeks).

**Results:**

Slightly over one third of infants (35.76%) had RIWG (total *N* = 330). Hypertension in pregnancy and/or pre-existing hypertension (OR 2.16, 95%CI 1.05–4.45) and birthweight Z score (OR 0.42, 95%CI 0.28–0.63) were associated with RIWG. Similarly, maternal hypertension was positively and birthweight Z score was negatively associated with WAZ score change (Coeff = 0.37, 95%CI 0.08–0.70; Coeff=-0.47, 95%CI -0.61-(-)0.34 respectively). LTL at birth was also positively associated with WAZ score change (Coeff = 0.41, 0.04–0.78). Birthweight Z score was associated with WAZ score at 6 months (OR 0.55795%CI 0.43–0.71) as was maternal hypertension (Coeff = 0.36 (95% CI, 0.06–0.66)).

**Conclusions:**

In our primarily Latinx cohort, there were similar risk factors for RIWG and WAZ change from birth to 6 months of age. Pre-existing and/or gestational hypertension was associated with RIWG and WAZ change. Longer LTL at birth may be a marker of greater WAZ change.

**Supplementary Information:**

The online version contains supplementary material available at 10.1186/s12884-025-08213-8.

## Impact Statement

Higher weight for age Z score (WAZ) change from birth to 6 months of age is associated with childhood obesity risk. There are few correlates of higher WAZ change, particularly among term infants. Our study highlights maternal hypertension and neonatal leukocyte telomere length (LTL) as predictors of WAZ change in Latinx infants.

## Background

### Childhood obesity

Childhood obesity impacts all demographic, racial and ethnic groups in the United States. In the past thirty years, the prevalence of obesity in the US has almost doubled in children and tripled in adolescents [1]. Children with overweight and obesity in childhood are more likely to have overweight or obesity in adulthood [2]. Obesity in childhood has a significant impact on psychological and physical health including risk for diabetes mellitus, coronary heart disease, hypertension, and hyperlipidemia [3]. Latinx children are more likely to be overweight and obese than Black, non-Hispanic White and Asian children in the United States. The Centers for Disease Control (CDC) reported that 26.2% of Latinx children ages 2–17 had obesity compared with 24.8% of non-Hispanic Black, 16.6% among non-Hispanic White and 9.0% among non-Hispanic Asian children using data from the National Health and Nutrition Examination Survey (2017–2020) [4].

### Rapid infant weight gain

Ideally, childhood obesity could be prevented early in life childhood as once children have obesity it is difficult to reverse as there are few successful interventions to treat obesity [5, 6]. Rapid infant weight gain (RIWG) which is defined as a change of more than 0.67 SDs in WAZ between two time points during infancy (up to 2 age years) [7, 8], is a significant early life risk factor for future childhood obesity as indicated in our previous work and that of others [9]. The exact mechanism of RIWG remains poorly described [10], but infants with RIWG, particularly from 0 to 6 months of age have greater future central fat deposition, fat accumulation and greater risk for future obesity [11, 12]. RIWG, using heterogeneous definitions of excess weight change in the first 2 years of life is more strongly associated with future obesity among Latinx and Black racial and ethnic groups in contrast with non-Hispanic Whites and as such, targeting RIWG for intervention may provide additional benefits to communities disproportionately impacted by obesity [13–15].

### Preterm birth and known risk factors for rapid infant weight gain

Risk factors associated with RIWG include lower birthweight including small for gestational age (SGA) births, male sex, formula feeding versus breastfeeding [7] and early added sugar consumption [16]. Other risk factors include lower gestational age at birth [17] and specific genetic single nucleotide polymorphisms (SNPs) associated with obesity [18]. A recent Australian study found a positive association between gestational hypertension and accelerated weight gain from birth until two years of age but no studies to our knowledge have evaluated the association between gestational and pre-existing hypertension and RIWG at 6 months of age, the time period of accelerated gain associated with the greatest risk for future obesity [19, 20].

The trajectory of weight gain in infancy differs for infants who were born preterm (< 37 weeks gestation) compared with those who were born at term with higher rates of RIWG particularly among preterm infants who are born late preterm (34-<37 weeks) [21]. The relationship between RIWG and obesity in preterm infants appears to be independent of birthweight status with a systematic review and meta-analysis finding no difference in outcomes between appropriate for gestational age (AGA) and SGA preterm infants [22]. RIWG in preterm infants likely has different metabolic pathways and possibly a different definitional framework for rapid and excessive gain as some catch up growth is necessary for healthy growth for preterm infants [22].

### Leukocyte telomere length

Additional biomarkers at birth that are associated with risk for RIWG in the first 6 months of life would be helpful so as to target interventions for high-risk infants. Specifically, known modifiable risk factors such as feeding type and schedule could be presented in a more intensive format to families with heightened risk [7, 16]. Our group has previously investigated the association between leukocyte telomere length (LTL), the non-coding nucleotide sequence (TTAGGG) that form a cap at the top of chromosomes to protect the chromosome from damage and degradation, and childhood obesity finding that shorter LTL measured in early childhood is associated with obesity in middle childhood [23] and that LTL at birth is associated with obesity at 12 months of age [24]. Inflammation and reactive oxygen species have been shown to adversely impact LTL in many adult and pediatric studies [25] and chronic disease studies have found that shorter LTL can predict incident metabolic disease including type 2 diabetes mellitus through a pancreatic ß-cell senescence and changes in adipocyte metabolic profiles [26]. Studies with animals suggest that the disruption of telomeric protein increases risk for early onset of obesity and abdominal fat suggesting that telomeres may be involved in metabolic processes [27]. Once LTL reaches a critical length, shortened telomeres are pro-inflammatory and could stimulate an obesity and metabolic disease cascade [28].

To our understanding, no study has investigated the possible role of LTL at birth and risk of RIWG at the 6 month timepoint although a few studies have assessed the association between early obesity and subsequent LTL or LTL change. Accelerated LTL loss from 3 month to 2 years was associated with greater fat mass % from 3 to 6 months of age [29] and our previous study found that obesity at 6 months was associated with shorter LTL at 3–4 years of age [30].

In this large study of primarily Latinx children recruited from two hospitals at San Francisco at birth, we evaluate risk factors for RIWG and overall patterns of weight gain in the first 6 months of life. We chose these outcomes based on the importance of early indicators for future childhood obesity risk [9]. In particular, we evaluate the role of gestational hypertension and/or pre-existing hypertension and LTL as possible risk factors for accelerated infant weight gain in the first 6 months of life as well as the associative relationship between gestational and/or pre-existing hypertension, LTL and infant weight trajectories in the first 6 months. We hypothesized that shorter LTL at birth adjusting for gestational age and exposure to gestational and/or pre-existing hypertension would be associated with greater risk for RIWG and greater WAZ at 6 months.

## Methods

### Cohort and inclusion, exclusion criteria

Data were collected from the Telomere at Birth (TAB) cohort, a longitudinal study of mothers and babies recruited in the immediate postnatal period (at 12-24 hours postpartum)at UCSF Benioff and Zuckerberg San Francisco General (ZSFG) Hospitals from August 2016 to March 2020 to assess how intrauterine exposures impact LTL and whether LTL can be a risk factor for postnatal growth trajectories. Maternal pre-existing and gestational hypertension was an additional predictor of interest. Eligibility criteria for maternal enrollment included English or Spanish speaking based on self-report, no history of active illicit drug use and plans to stay in the San Francisco Bay Area for the foreseeable future. Inclusion criteria for infant enrollment included the following: >32 weeks gestational age, singleton birth, no contraindications for breastfeeding, in the newborn nursery or intensive care nursery without any imminent surgery or chronic disease condition including possibility of HIV infection or history of blood transfusions.

### Study procedures

Eligible study participants were screened via electronic medical record review in both hospitals and subsequently approached prior to 24-hours postpartum. Only participants that met all inclusion criteria were approached for enrollment. Bilingual research coordinators explained all study procedures to potential participants in language of choice (Spanish or English) and participants signed written consents for their and their children’s participation. Participants were subsequently interviewed using a one-page questionnaire to collect information on exposures in pregnancy including dietary ones (specifically sugar sweetened beverage (SSB), defined as all sodas, fruit juices and sweetened teas, and 100% fruit juice intake during pregnancy), smoking/second hand smoke exposure and weight gain in pregnancy. SSB intake in pregnancy was targeted given previous data suggesting an association between intake and childhood obesity [31] and our previous data suggesting that early child SSB intake including flavored milks may adversely impact LTL in children, likely by increasing overall inflammation and metabolic disease [30, 32]. Self -reported medical conditions were also collected and the medical record was evaluated to assess infant anthropometrics, gestational age and any maternal or infant medical diagnoses including pre-existing and/or gestational hypertension. Maternal pre-pregnancy body mass index (BMI) was calculated as weight (kilograms)/height (meters)^^2^ with underweight defined as having a BMI below 18.5, normal as 18.5-<25, overweight as 25-<30 and obese as > = 30 using CDC guidelines [33]. Appropriate maternal weight gain in pregnancy was ascertained using the American College of Obstetrics and Gynecology (ACOG) recommendations as per the Institute of Medicine’s guidelines [34]. Newborn birthweight and length were ascertained at UCSF and ZSFG using standard digital scales and standard tape measurements. All data was uploaded into RedCap.

At 4–6 weeks of age, participants were contacted telephonically to collect information on infant feeding including breastfeeding, formula feeding and any introduction of non-milk liquids or solids. Exclusive breastfeeding was defined using the World Health Organization’s (WHO) definition as that of feeding only breastmilk without the addition of any milk substitutes or other non-milk liquids or solids [35]. Our questionnaires have been previously used to collect information in Spanish and English-speaking women in similar populations of postpartum women in San Francisco [30, 36]. At 6 months of age (or closest time point), infant’s weight and length were collected by in-person measurements using standard digital scales and tape measurements, self-reported measurements via telephonic interview or extraction from the medical record. For the self-reported measurements, parents were asked to report weight and length from a 6-month doctor’s visit and the visit was verified by health summary visit information (parents sent us email copies of growth charts or pediatric visit take home information). If the 6-month visit was not available, the visit with the closest age to 6 months was used. Approximately 50% of measurements were self-reported or extracted from the medical record and all were confirmed via medical chart review. To transform infant weights and lengths into Z scores, we used the CDC’s 2000 Growth Curves and exact age of child’s measurement and child sex was used to get age and sex specific WAZ score [37]. To calculate change in WAZ score we subtracted the WAZ score at birth from the WAZ score at the approximate 6-month timepoint. All study procedures were approved by the Institutional Review Board (IRB). Informed signed written consent was obtained from all subjects and/or their legal guardians.

### Leukocyte telomere length measurements

Infant LTL was collected as part of the study protocol. At the same time as the California Newborn Genetic screen, 5 drops of blood were collected via heelstick from newborns on Whatman dried bloodspot (DBS) cards. Cards were subsequently stored at the Clinical and Translational Sciences Institute (CTSI) at UCSF Benioff and ZSFG hospitals in (-) 80 F freezers. Samples were subsequently sent to the Blackburn laboratory where LTL was assessed in batch. LTL is reported as a T/S ratio, or the ratio of telomeric product to a single-copy gene product, as determined by quantitative polymerase chain reaction (PCR). The exact methodology used has been previously described [38, 39].

DNA was extracted from the DBS on Whatman 903 cards with the QIAamp DNA Investigator kit (QIAGEN cat# 56504) and eluted in 50 ul ATE buffer. The DBS were collected between August 2016 to March 2020, stored at −80 °C and batch-extracted in November 2020. Deoxyribonucleic acid (DNA) was stored at −80 °C and LTL assays were performed between November to December of 2020. A 3-fold serial dilution of a commercial human genomic DNA (Sigma-Aldrich, cat#11691112001) containing 26, 8.75, 2.9, 0.97, 0.324 and 0.108ng of DNA was included in each PCR run as the reference standard. The quantity of targeted templates in each sample was determined relative to the reference DNA sample by the maximum second derivative method in the Roche LC480 program. The reaction was carried out in a Roche LightCycler 480 in 384-well plates, with triplicate wells for each sample. Dixon Q test was used to exclude outliers from the triplicates. The average of the telomere (T) and single copy (S) triplicate wells after outliner removal was used to calculate the T/S ratio for each sample. The same reference DNA was used for all PCR runs. The PCR efficiencies of the T and S reactions were 88.4%±4.1% and 91.4%±3.9% respectively.

The average inter-assay coefficient of variation (CV) for this study was 2.3 ± 1.5%. Intra-class correlation (ICC) of repeat extractions of 46 dried blood spot samples T and S reactions from this study was 0.883 (CI [0.766–0.942]).

### Statistical analysis

Tests for normality including Shapiro-Wilk were conducted for continuous outcomes including infant WAZ at approximately 6 months and WAZ change from 0 to 6 (or closest measurement) months of age. As these outcomes were normally distributed, parametric tests were used to assess statistical significance including student t-tests for dichotomous variables and Pearson’s correlation coefficient for continuous ones (r). RIWG was defined as weight gain > 0.67 SD change from WAZ at birth to 6 months of age using as close to the 6-month time point as possible with mean time of measurement for the cohort as 6.39 ± 1.53 months of age. Analyses of covariates associated with RIWG were performed using chi-square tests for categorical variables and unpaired t-tests for continuous, normally distributed variables and Wilcoxson signed rank test (non-parametric test) for variables that did not have a normal distribution. Multivariable logistic regression and linear regression models were constructed using variables with a significance of *p* ≤ 0.10 in bivariate analysis to reduce the likelihood of a Type II error and variables with biological plausibility for association with RIWG, WAZ change and WAZ at 6 months including maternal age, child sex, child LTL and gestational age at birth. Multivariable models had different total N’s based on missing data for maternal and child variables and slightly different variables based on significance levels in bivariate analysis. Along these lines, we tested the possibility that different variables could be associated with alternative patterns of weight gain during the first 6 months of life. We did not include variables that were highly correlated with each other in the same models including maternal BMI and BMI weight category or weight gain in pregnancy and weight within/outside the ACOG guidelines. We also followed statistical guidelines of approximately 10 events per variable for regression models [40].

Models were restricted to > = 37 weeks gestation and < 37 weeks gestation to assess whether results differed for term infants alone given the different growth trajectories that preterm and term infants have from each other. These findings were presented only in the text and not in tabular form. We also conducted a sensitivity analysis of birthweight Z score for preterm infants using Fenton growth curves [41] and compared the findings using these curves with our initial calculation using CDC curves to assess RIWG. All analyses were conducted using Stata 15.0.

## Results

There were 330 mother and infant pairs who completed the 6-month follow-up from delivery through weight assessment at 6 months of age. The mean age at 6 months measurement was 6.33 ± 0.92. months (Table 1). Slightly over one third of the cohort (35.61%) had RIWG; the percentage was also 35.61% using the Fenton growth curves for preterm infants (41/330; 12.42%). The mean WAZ change from 0 to approximately 6 months was 0.26 ± 1.06 (Table 1 supplement) but slightly less using the Fenton curves for the preterm infants 0.16 ± 1.04 (results not shown).

**Table 1:** Maternal, Paternal and Infant Characteristics in Relation to Rapid Infant Weight Gain* at 6 Months of Age

Variable	% (*N*/Total) or mean ± SD)	RWIG % (*N*/Total)	No RIWG % (*N*/Total)	*p* value
Total Cohort, count	330	35.76 (118/330)	64.24 (212/330)	
Age at approximately				
6 months (in months)	6.33± 0.92			
Maternal and Paternal Demographics				
*Maternal Education*				0.06
Less than high school	13.13 (43/328)	17.80 (21/118)	10.48 (22/210)	
High school or more	86.89 (285/328)	82.2 (97/118)	89.52 (188/210)	
*Marital Status*				0.48
Married/Living with partner	92.07 (302/328)	90.68 (107/118)	92.86 (195/210)	
Single/Other	7.93 (28/328)	9.32 (11/118)	7.14 (15/210)	
*Maternal Race/Ethnicity*				
White	24.4 (80/329)	24.58 (29/118)	24.17 (51/211)	0.80
Asian/Pacific Islander	8.51 (28/329)	6.78 (8/118)	9.48 (20/211)	
Black	1.22(4/329)	1.69 (2/118)	0.95 (2/211)	
Latina	65.96(217/329)	66.95 (79/118)	65.40 (138/211)	
*Maternal Ethnicity*				0.20
*Maternal Latina Ethnicity*				
Mexican/Central American	51.21 (169/330)	55.93(66/118)	48.58 (102/212)	
Not Mexican/ Central American	48.79 (161/330)	44.07 (52/118)	51.42 (109/212)	
*Maternal Latina Ethnicity*				
Caribbean/South American (SA)/Spain Portugal	16.36 (54/330)	15.25 (18/118)	16.98 (36/212)	0.68
Not Caribbean/SA/ Spain/Portugal	83.64 (276/330)	84.75 (100/118)	83.02 (176/212)	
*Paternal Latino Ethnicity*				0.02
Mexican/Central American (CA)	35. 15 (116/330)	43.22 (51/118)	30.66 (65/212)	
Not Mexican/CA	64.85 (214/330)	56.78 (67/118)	69.34 (147/212)	
*Paternal Latino Ethnicity*				
Caribbean/SA/Spain/Portugal	7.88 (26/330)	6.78 (8/118)	8.49 (18/212)	0.58
Not Caribbean/SA/Spain/Portugal	92.12 (304/330)	93.22 (110/118)	91.51 (194/212)	
Any parent - Mexican/CA ethnicity				
No	45.15 (149/330)	37.29 (44/118)	49.53 (105/212)	0.03
Yes	54.85 (181/330)	62.71 (74/118)	50.47 (107/212)	
*Maternal Age, years*	32.18± 5.45	31.56± 5.68	32.52± 5.29	0.12
*Maternal Age, years*				0.13
<20	2.43 (8/329)	4.27 (5/117)	1.42 (4/217)	
>=20 -<30	23.71 (78/329)	25.64 (30/117)	22.64 (48/217)	
>=30-<35	35.26 (116/329)	38.46 (45/117)	33.49(71/212)	
>=35	38.60 (127/329)	31.62 (37/117)	42.45 (90/212)	
*Age of Menarche, years*	12.61± 1.59	12.65± 1.51	12.59± 1.64	0.76
<12	26.22 (86/328)	22.88 (27/118)	28.10 (59/210)	0.38
12	19.51 (64/328)	17.80 (21/118)	20.48 (43/210)	
>12	54.27 (178/328)	59.32 (70/118)	51.43 (108/210)	
Maternal Health History				
*Maternal Gestational and/or Pre-existing Diabetes Mellitus*				0.77
Any diabetes	21.21 (70/330)	20.34 (24/118)	21.20 (46/212)	
No diabetes	78.79 (260/330)	79.68 (94/118)	78.30 (166/212)	
*Maternal Gestational and/or Pre-existing Hypertension*				<0.01
Any hypertension	16.97 (56/330)	25.42 (30/118)	12.26 (26/212)	
No hypertension	83.03 (274/330)	74.58 (88/118)	87.74 (186/212)	
*Maternal Smoking/Secondhand Smoke Exposure*				0.67
Any smoke exposure	9.39 (31/330)	8.47 (10/118)	9.91 (21/212)	
No smoke exposure	90.61 (299/330)	91.53 (108/118)	90.09 (191/212)	
*Sugar Sweetened Beverage (SSB) intake, cups*				
SSB intake, cups/week	2.42± 3.73	2.85± 4.18	2.18± 3.44	0.12
No-<1 SSB cup/week >=1/week	45.73 (150/328)	40.17 (47/117)	48.82 (103/211)	0.13
100% Fruit Juice, cups/week	54.27 (178/328)	59.83 (70/117)	51.18 (108/211)	
100% Fruit Juice, cups	2.85+/-5.22	3.53+/-5.95	2.47+/-4.74	0.08
				0.21
0 cups/week	35.17 (115/327)	37.67 (81/215)	37.62 (79/210)	
>0 cups/week	64.83 (212/327)	62.33 (134/215)	62.38 (131/210)	
*Mental illness in Pregnancy*				0.47
None	85.76 (283/330)	83.90 (99/118)	86.79 (184/212)	
Yes	14.24 (47/330)	16.10 (19/118)	13.21 (28/212)	
Parity (continuous)	1.80± 0.92	1.84± 1.05	1.81± 0.93	0.60
Parity (categorical)				0.29
1	46.87 (153/329)	47.86 (56/117)	45.75 (97/212)	
2	32.24 (106/329)	27.35 (32/117)	34.91 (74/212)	
>=3	20.90 (70/329)	24.79 (29/117)	19.34 (41/212)	
*Medication(s) for Depression/Anxiety*				0.74
Yes	9.93(30/302)	9.17 (10/109)	10.15 (20/197)	
No	90.07 (272/302)	90.83 (99/109)	89.85 (177/197)	
* Pre-Pregnancy BMI*	26.18± 6.57	26.33±7.02	26.10±6.32	0.77
Category				0.24
Underweight (<18)	1.91 (6/314)	3.57 (4/112)	0.99 (2/202)	
Normal (>=18 - <25)	51.59 (162/314)	47.32 (53/112)	53.96 (109/202)	
Overweight (>=25 -<30)	24.20 (76/314)	23.21 (26/112)	24.75 (50/202)	
Obese (>= 30)	22.29 (70/314)	25.89 (29/112)	20.30 (41/202)	
*Maternal Gestational Weight Gain, lbs*	27.57 ± 11.32	25.92± 10.59	28.46± 11.62	0.05
*Maternal Gestational Weight Gain *				
>40 lbs	12.96 (42/324)	12.28 (14/114)	13.33 (28/210)	0.79
<=40 lbs	87.04 (282/324)	87.72(100/114)	86.67 (182/210)	
*American College of Obstetrics and Gynecology (ACOG) Gestational Weight Gain*				
Below	22.83 (71/311)	28.18 (12/110)	19.90 (40/201)	0.23
Within	42.44 (132/311)	30.91 (34/110)	43.28 (87/201)	
Over	34.73 (108/311)	40.91 (45/110)	36.82 (74/201)	
Child-Specific				
*Gestational Age, weeks*	38.79± 1.80	38.04± 2.07	39.21± 1.48	<0.01
*Gestational Age, category*				<0.01
32-<35 wks	4.24 (14/330)	9.32 (11/118)	1.42 (3/212)	
≥35 wks and <37 wks	8.18 (27/330)	13.56 (16/118)	5.19 (11/212)	
≥37 wks	87.58 (289/330)	77.12 (91/118)	93.40 (198/212)	
*Child Sex*				0.27
Female	48.48 (160/330)	52.54 (62/118)	46.23(98/212)	
Male	51.52 (170/330)	47.67 (56/118)	53.77 (114/212)	
Apgar (5 mins)	8.79+/-0.65	8.73+/0.68	8.82+/-0.63	0.27
*Apgar (5 mins)*				0.09
<9	15.81 (52/329)	20.34 (24/118)	13.27 (28/211)	
≥9	84.19 (277/329)	80.0 (94/118)	86.73 (183/211)	
*Birth Type*				0.62
C-Section	23.78 (78/328)	22.22 (26/117)	24.64 (52/211)	
Vaginal	76.22 (250/328)	77.78 (91/117)	75.36 (159/211)	
*Child Birthweight, Z score*	-0.32± -1.00	-0.85± 0.86	-0.02± 0.95	<0.01
*Child Birthweight, kg*	3.27± 0.54	2.98± 0.50	3.43±0.50	<0.01
*Low Birthweight (<2500 g)*				<0.01
Yes	6.97 (23/330)	15.00 (18/118)	2.36 (5/212)	
No	93.03 (307/330)	84.75 (100/118)	97.64 (207/212)	
*Macrosomic (>4000 g)*				<0.01
Yes	6.36 (21/330)	0.0 (0/118)	9.91 (21/212)	
No	93.64 (309/330)	100.0 (118/118)	90.09 (191/212)	
*Leukocytem Telomere Length (LTL) T/S ratio*	2.02± 0.28	2.04± 0.27	2.01± 0.29	0.44
*Breastfeeding (BF) at discharge*				
Mixed feeding	21.65 (71/328)	25.64 (30/117)	19.44 (41/211)	0.19
Exclusive breastmilk	78.35 (257/328)	74.36 (87/117)	80.57 (170/211)	
*Breastfed 6 weeks*				
Yes	88.60 (272/307)	87.27 (96/110)	89.34 (176/197)	0.59
No	11.40 (35/307)	12.73 (14/110)	10.66 (21/197)	
*Exclusively BF, 6 weeks*				0.33
Yes	60.91 (187/307)	57.27 (63/110)	62.94 (124/197)	
No	39.09 (120/307)	42.73 (47/110)	37.06 (73/197)	

* Rapid infant weight gain (RIWG) defined as change >0.67 SD in weight Z score from birth to 6 months

Other characteristics of the sample included the following: 86.89% of the mothers had at minimum a high school diploma and 92.07% were living with a partner or married. Overall, 65.96% of mothers reported Latina ancestry (Table 1).

For the infants, the mean gestational age of the cohort was 38.79 ± 1.80 weeks with 12.42% of the cohort < 37 weeks of age (preterm). Mean child birthweight was 3.26 ± 0.54 kg and 6.79% of the sample was low birth weight (< 2,500 g). Mean LTL in the sample population at birth was 2.02 ± 0.28 T/S units (Table 1).

### Variables associated with RIWG

A higher percentage of mothers with less than a high school education had infants with RIWG compared to those with more education although the results did not meet statistical significance (17.80% versus 13.13%, *p* = 0.06 Table 1). Paternal ancestry was also associated with RIWG with 43.22% of fathers with Mexican/Central American ancestry having infants with RIWG compared to 30.66% without (*p* = 0.02). Similarly, having any or both parents with Mexican/Central American ancestry was associated with increased risk for RIWG (62.71% versus 50.47%; *p* = 0.03). Mothers with pre-existing and/or gestational hypertension had greater risk for RIWG (25.42% versus 12.26%; *p* < 0.01) as did those who gained less weight in pregnancy (25.92+/−10.59 versus 28.46+/−11.62 pounds, *p* = 0.05) although the results did not meet the threshold for statistical significance (Table 1). Other maternal demographics including maternal race/ethnicity, age and other health history variables were not associated with RIWG (Table 1).

Gestational age at birth differed between those infants with RIWG compared to those without (38.79 ± 1.80 versus 38.04 ± 2.07 weeks; *p* < 0.01) with a higher number of preterm infants having RIWG compared with those born at term (22.57% versus 6.61%; *p* < 0.01). The mean birthweight of infants with RIWG was lower than those without RIWG (2.98 ± 0.50 kg versus 3.43 ± 0.50 kg, *p* < 0.01) with a higher number of LBW infants with RIWG (15.0% versus 2.36%, *p* < 0.01; Table 1). Other child-specific variables including sex and breastfeeding status were not associated with RIWG (Table 1).

### Variables associated with WAZ change 0 to 6 months

There was some consistency between variables associated with RIWG and increasing WAZ change from 0 to 6 months of age including lower levels of maternal education, having a parent with Mexican/Central American ancestry and maternal gestational and/or pre-existing hypertension (Table 1 supplement). Similar to risk for RIWG, women who had higher levels of maternal weight gain in pregnancy had infants with lower WAZ changes (Table 1 supplement). Women with greater SSB consumption (> 1 cup/week compared to <=1 cup/week) had infants with greater WAZ changes. Higher infant birthweight Z scores and birthweight (kg) were also associated with lower WAZ changes and greater LTL was associated with greater changes (Table 1 supplement). Lastly, LTL at birth was associated with greater WAZ changes from birth to 6 months of age (Table 1 supplement). Other maternal and child demographic and health variables were not associated with WAZ score changes (Table 1 supplement).

### Variables associated with WAZ at 6 months of age

Fewer variables were associated with greater WAZ scores at 6 months of age. The following infant variables were positively associated with WAZ at 6 months; gestational age , vaginal birth (versus C-section), birthweight (kg) and birthweight Z score were positively associated with WAZ at 6 months (Table 1 Supplement). Infants exclusively breastfed at 4–6 weeks of age had greater WAZ scores at 6 months (Table 1 Supplement).

### Multivariable models

#### RIWG

**Fig. 1:**
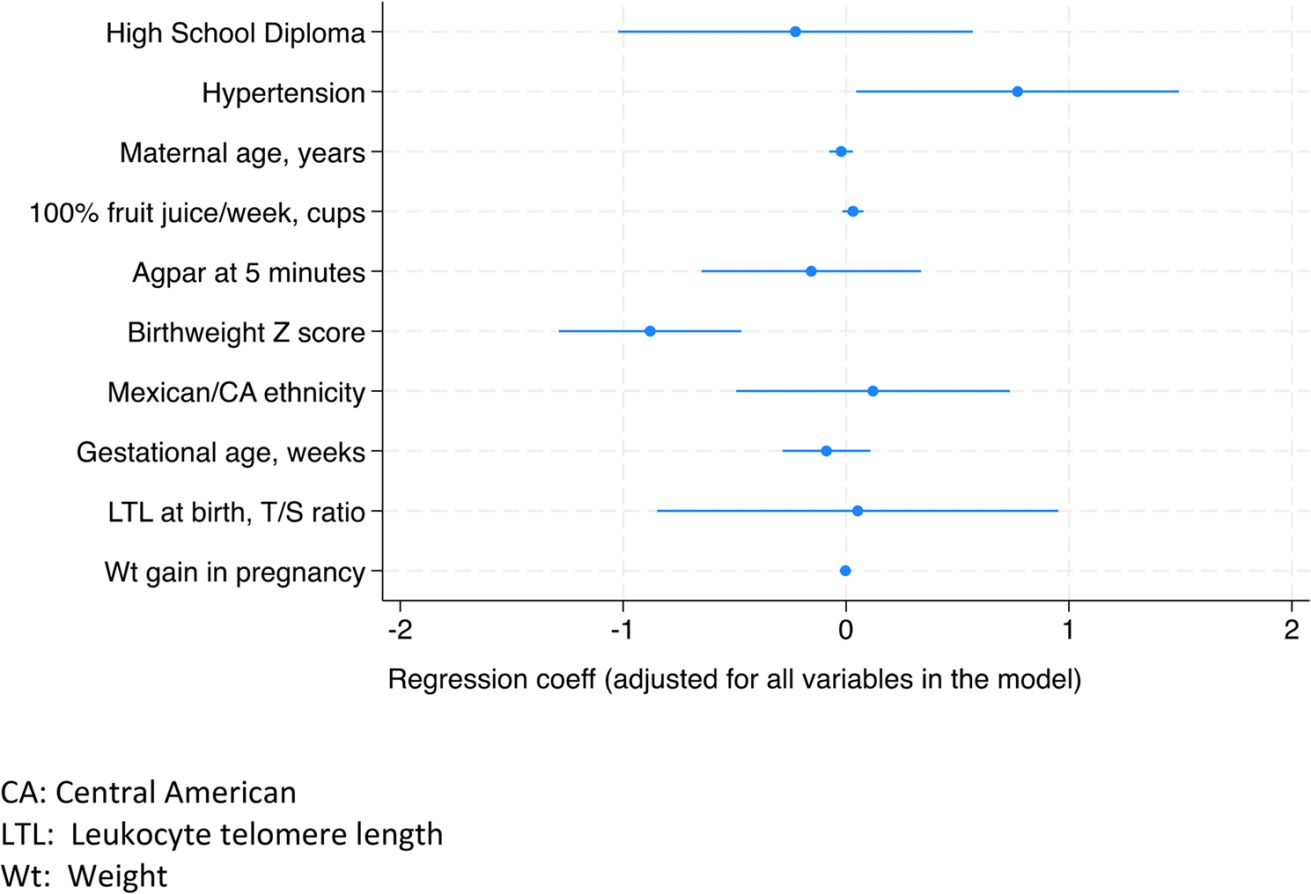
Maternal and Infant Risk Factors for Rapid Infant Weight Gain, Multivariable Model

In a model of RIWG including maternal education level (high school diploma and above versus lower), maternal hypertension (gestational and/or pre-existing, yes versus no), maternal age (years), 100% juice consumption per week (cups), Apgar at 5 min, birthweight Z score, any parent of Mexican or Central American origin (yes versus no), gestational age, infant LTL at birth (T/S ratio) and weight gain in pregnancy (lbs), two variables were associated with RIWG: gestational and/or pre-existing hypertension (Odds Ratio (OR) 2.16, 95%CI 1.05–4.45) and birthweight Z score (OR) 0.42, 95%CI 0.28–0.63) (Table 2; Fig. 1). When we restricted the analysis to > = 37 weeks, or excluded preterm infants, the variables that were statistically significant remained the same (results not shown). In an analysis that included preterm infants alone, neither variable was associated with RIWG.

**Table 2:** Multivariable Risk Factors for Rapid Infant Weight Gain (RIWG) from Birth to 6 Months of Age (*n*=311)^+^

Variable	OR, 95%CI	*p* value
High school diploma	0.80 (0.36, 1.77)	0.58
Maternal hypertension*	2.16 (1.05, 4.45)	0.03
Maternal age, years	0.98 (0.93-1.03)	0.41
100% Fruit juice per week in pregnancy, cups	1.03 (0.98, 1.08)	0.20
Apgar at 5 minutes	0.86 (0.51, 1.40)	0.53
Birthweight Z Score	0.42 (0.28, 0.63)	<0.01
Any parent with Mexican/CA Ancestry	1.13 (0.61, 2.08)	0.70
Gestational age, weeks	0.92 (0.75, 1.11)	038
Leukocyte telomere length at birth (T/S)	1.05 (0.43, 2.59)	0.91
Weight gain in pregnancy (lbs)	1.00 (0.97, 1.02)	0.84

*Pre-existing or gestational

+Total *N* is based on complete data as participants with missing data are not included

#### WAZ Score Change

**Fig. 2:**
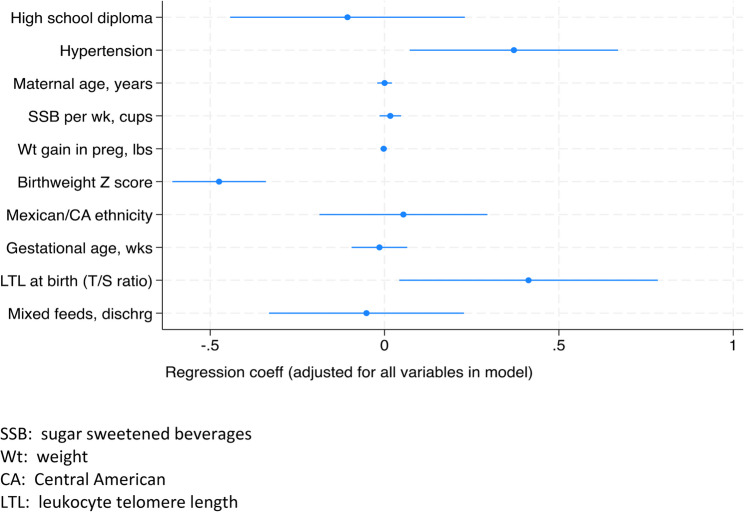
Risk Factors for WAZ Change, Multivariable Model

In a model of WAZ score change including the same variables as in the RIWG model except replacing SSB intake for 100% fruit juice consumption (cups/week), adding mixed breastfeeding at hospital discharge and removing Apgar at 5 min, 3 variables were significant (Table 3). Pre-existing and/or gestational hypertension was associated with greater WAZ score change (Coeff = 0.37, 95%CI 0.08–0.70) and birthweight Z score was negatively associated with Z score change (Coeff=−0.47, 95%CI −0.61-(-)0.34; Table 3; Fig. 2). Newborn LTL was positively associated with Z score change (Coeff = 0.35, 95%CI −0.02-0.71; Table 3; Fig. 2). Restricting the sample to those that infants that were born > = 37 weeks, the effect size between LTL at birth and WAZ score change was stronger (Coeff = 0.60, 95%CI 0.16–1.04; results not shown). Maternal gestational and/or pre-existing hypertension and birthweight Z score were still significantly positively associated with WAZ score change. We did not find any variables that were statistically significant when we restricted the multivariable analysis to the preterm infant sample alone.

**Table 3:** Multivariable Linear Regression for WAZ Score Change from Birth to 6 Months (*n*=311)^+^

Variable	Coefficient (95%CI)	*p* value
High school diploma	-0.11 (-0.44, 0.23)	0.54
Maternal hypertension*	0.37 (0.08, 0.70)	0.02
Maternal age, years	0.0002 (-0.02, 0.02)	0.99
Sugar-sweetened beverage (SSB)		
consumption per week in pregnancy, cups	0.02 (-0.01, 0.05)	0.29
Weight gain in pregnancy, lbs	-0.002 (-0.01, 0.007)	0.62
Birthweight Z score	-0.47 (-0.61, -0.34)	<0.01
Any parent with Mexican or Central		
American ethnicity	0.05 (-0.19, 0.30)	0.56
Gestational age, weeks	-0.01 (-0.09, 0.07)	0.72
Leukocyte telomere length at birth (T/S)	0.41 (0.04, 0.78)	0.03
Mixed breastfeeding at discharge	-0.05 (-0.33, 0.23)	0.72

*Pre-existing or gestational

+Total *N* is based on complete data as participants with missing data are not included

#### WAZ Score at 6 Months

**Fig. 3:**
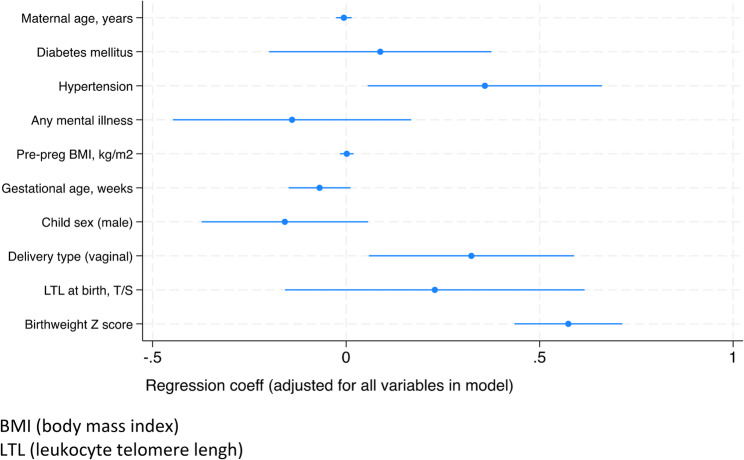
WAZ at 6 Months, Multivariable Model

In a model of WAZ score at 6 months including maternal age (years), any diabetes mellitus (including pre-existing or gestational), any mental illness in pregnancy, pre-pregnancy BMI (kg/m2), delivery type, and the same infants specific variables as in the WAZ score change model, 3 variables were associated with WAZ score at 6 months. Maternal gestational and/or pre-existing hypertension (OR 0.36, 95%CI 0.06–0.66; Table 4; Fig. 3) and birthweight Z score were positively associated with WAZ score at 6 months (OR 0.57, 95%CI 0.43–0.71 Table 4; Fig. 3). Vaginal delivery was also associated with greater WAZ score at 6 months (OR 0.23, 95%CI 0.06-0.59; Table 4, Fig. 3). Restricting the sample to term babies alone, birthweight Z score was positively associated with WAZ at 6 months and LTL at birth was positively associated with WAZ score at 6 months (OR 0.50, 95%CI 0.06–0.94; *p* = 0.03; results not shown). For preterm infants, birthweight Z score but not LTL was similarly associated with WAZ at 6 months of age (results not shown).

**Table 4:** Multivariable Risk Factors for WAZ Score at 6 Months (*n*=303)

Variable	Coefficient (95%CI)	*P* Value
Maternal age, years	-0.006 (-0.03, 0.01)	0.57
Maternal diabetes*	0.09 (-0.20, 0.38)	0.55
Maternal hypertension*	0.36 (0.06, 0.66)	0.02
Any mental illness in pregnancy	-0.14 (-0.45, 0.17)	0.37
Maternal pre-pregnancy		
Body Mass Index (BMI)	0.002 (-0.02-0.02)	0.84
Gestational age, weeks	-0.07 (-0.15, 0.01)	0.09
Child sex (Male)	-0.16 (-0.37, 0.06)	0.15
Delivery type (C-section versus Vaginal)	0.23 (0.06-0.59)	0.02
Leukocyte telomere kength at birth (T/S)	0.23 (-0.16, 0.62)	0.25
Birthweight Z score	0.57 (0.43, 0.71)	<0.01

*Pre-existing or gestational

+Total *N* is based on complete data as participants with missing data are not included

## Discussion

In this primarily Latinx birth cohort from two hospitals in San Francisco, we confirmed the strong association between higher birthweight Z scores and lower risk of RIWG as demonstrated in previous pooled meta-analyses [17]. We also found that pre-existing and/or gestational hypertension was strongly associated with RIWG, greater WAZ change from birth to 6 months of age and WAZ at 6 months. Previous studies have found that gestational hypertension or increased maternal blood pressure measurements in the postpartum period are associated with greater risk for childhood obesity [11, 42, 43] and greater WAZ change from 1 to 5 years of age [44]. However, our study is the first to our knowledge which confirms an association between gestational and/or pre-existing hypertension and RIWG defined as excessive gain from birth to 6 months of age. The uniform association between maternal hypertension and early markers of future obesity risk (RIWG, WAZ change and WAZ at 6 months) attest to the importance of blood pressure control in pregnancy and further exploration of pathways associated with maternal blood pressure alterations and infant weight trajectories. We also found that maternal pre-existing and/or gestational hypertension was associated with infancy weight outcomes for births at all gestational ages as well as when the sample was restricted to term infants suggesting the importance of this clinical indicator for early life weight gain. As other studies have found that gestational hypertension exposures can impact obesity risk as late as the adolescent years, a focus on blood pressure control in gestational hypertension or pre-existing hypertension for women of reproductive age may be an essential target for pediatric obesity interventions [43].

We did not find any association with pre-existing and/or gestational diabetes mellitus and RIWG, WAZ from birth to 6 months or WAZ at 6 months of age. It is possible that maternal BMI category and weight gain in pregnancy were stronger indicators than maternal diabetes or gestational diabetes mellitus (GDM) as previous studies have found that the association between GDM and childhood obesity is attenuated when maternal obesity is accounted for [45].

### Leukocyte telomere length

Our study is also the first to find a positive association between newborn LTL and WAZ change from birth to 6 months of age. We did not find any association between LTL and RIWG but higher LTL was associated with greater WAZ changes from birth to 6 months.

There was also a trend towards longer LTL at birth in association WAZ at 6 months in term infants alone. A previous study found that longer LTL was associated with greater lean infant body mass at 12 months of age in infants of all gestational ages [46] including SGA, appropriate for gestational age (AGA) and large for gestational age (LGA). At 2 weeks of age, longer LTL was also associated with fat mass in addition to lean body mass in this earlier study; however, we did not differentiate between fat and lean body mass in infants for our study.

Our findings of a positive association between newborn LTL and WAZ change at 6 months are in contrast to what we hypothesized which was a negative association between newborn LTL and RIWG. Our previous study with this same population group but at 12 months of age found a negative association between LTL at birth and obesity and WAZ at 12 months [24]. It is possible that longer LTL may be associated with healthy growth and weight gain for infants in the first 6 months until a certain threshold is reached but that there may be a negative association between LTL and RIWG. Alternatively, there may be a different threshold for association between LTL and RIWG for term versus preterm infants. Preterm infants or infants born SGA are at a greater risk for obesity from RIWG [22, 47] but also require a certain amount of catch up growth for healthy metabolism [48]. This may be why we only saw an association between LTL and WAZ at 6 months in term infants because patterns of weight gain in the first 6 months differ significantly between preterm and term infants with much of the catch growth happening in the first year of life [49]. The fat versus lean mass component of growth between preterm and term infants also differs.

Other studies have found that birth LTL is associated with fat free mass at 12 months in term infants [44] and preterm infants tend to have more fat mass than term infants during immediate postnatal growth [50, 51]. Fat mass accretion places infants at greater risk for future obesity. Our study was not powered to assess any association between LTL and RIWG among preterm infants and our overall sample size in those infants was also relatively small. Determinants of growth in the first 6 months may also vary in comparison with those at 12 months of age as our previous study did not find any mediation by WAZ score at 6 months in relation to LTL at birth and obesity at 12 months {24}.

WAZ change from birth to 6 months and WAZ at 6 months of age are important determinants of newborn health in the first year of life and the association with LTL may be an additional marker at birth that can help clinicians assess possible growth trajectories. Future larger studies are needed to better understand possible associations between LTL and RIWG compared with WAZ change in term and preterm neonates as well as the possible magnitude of any effect size and confidence intervals. Studies should also investigate the additive role that LTL at birth may play, particularly for term infants, in risk algorithm studies for healthy growth versus childhood obesity.

This may be particularly important as birthweight Z scores are negatively related to risk for RIWG and WAZ change yet positively correlated with WAZ at 6 months of age. As such, a higher or lower birthweight Z score alone is not sufficient to determine risk for future obesity; as our previous work has demonstrated, single variable studies are not optimized to determine risk for childhood obesity but rather a risk score that combines variables may have the strongest predictive value [9]. Additionally, future studies should investigate the possible predictive role of LTL as our study assessed only the associative role.

### Limitations

We assessed child growth status at 6 months of age using weight measurements alone in a relatively small cohort (*n* = 330) and future larger studies are needed to confirm associations and assess possible mediators of any proposed pathways. We also had child measurements that were collected at different timepoints close to the 6 month-mark (mean 6.39 ± 1.53 months) within a 4–8 month approximate range. Other studies have included additional measurements using dual X-ray absorptiometry (dexa) that would have helped provide more comprehensive determinants of body composition including % fat and lean body mass in children less than 12 months of age that could have provided additional insight on future risk for obesity [45]. While we collected postnatal data on maternal diet in pregnancy with a focus on SSB postnatally which may have introduced recall bias, additional comprehensive measures of maternal dietary intake should be assessed prospectively in future studies [52]. 

Our measurement at 6 months of age combined clinic visit measurements with research visit ones and ideally future studies should have only research associated measurements. Additionally, while we collected data on gestational and/or pre-existing hypertension that impacted pregnancy, we did not assess blood pressure control during pregnancy and medication usage patterns. Future studies that evaluate the role of maternal hypertension interventions on pediatric outcomes should include assessments of maternal blood pressure control to evaluate how these patterns may impact infant growth patterns. Lastly, we collected prenatal dietary data retrospectively which is subject to recall bias, and we did not include other aspects of maternal diet that could have impacted infant weight gain.

## Conclusions

Ours is the first study to find an association between gestational and/or pre-existing hypertension and infant growth patterns with a focus on RIWG in the first 6 months of life across neonatal gestational ages. Future obesity interventions with women of reproductive age should target maternal blood pressure control during pregnancy and prior to conception. We also found that infant LTL is associated with WAZ changes from birth to 6 months and possibly WAZ at 6 months for term infants. Future studies are needed with larger sample sizes including a greater number of preterm infants and better measures of body composition to understand the role that LTL at birth may play in discerning healthy growth patterns and for risk for obesity. A more comprehensive understanding of the pathways of risk, particularly for RIWG will help reduce the global burden of obesity for all children but may be particularly relevant for children of Latinx and Black race and ethnicity.

## Supplementary Information

Supplementary Material 1.

## Data Availability

Data will be available on individual request to the principal investigator (PI), Dr. Janet Wojcicki via email or written correspondence.

## Abbreviations

AGA: Appropriate for gestational age
BMI: Body mass index
CDC: Centers for Disease Control
DBS: Dried blood spot
DNA: Deoxyribonucleic acid
GDM: Gestational diabetes mellitus
LBW: Low birthweight
LGA: Large for gestational age
LTL: Leukocyte telomere length
RIWG: Rapid infant weigh gain
SGA: Small for gestational age
SNP: Single nucleotide polymorphisms
SSB: Sugar sweetened beverages
WAZ: Weight for age Z score
WHO: World Health Organization
