# Supplementary material for: Maternal hypertension and telomere length are associated with weight for age Z score change from birth to 6 months of age in a predominantly Latinx cohort
Source: BMC Pregnancy Childbirth. 2025 Nov 12;25:1195. doi: 10.1186/s12884-025-08213-8 (PMC12613348; doi:10.1186/s12884-025-08213-8)
Supplement: Supplementary file 1 — Supplementary Material 1. [file 12884_2025_8213_MOESM1_ESM.docx]

**TABLE 1 (Supplementary): Maternal, Paternal and Infant Characteristics in Relation to Weight-for-Age Z (WAZ) Score Change and WAZ Score at 6 Months of Age**

**Variable WAZ Score Change WAZ 6 mo Score**

**0-6 Mo (Mean ± SD) (Mean +/-SD) Corr. Coeff - Corr. Coeff and/or p- and/or p-**

**value value**

Total Cohort, 0.26 ± 1.06 -0.06± 1.05

at 6 months

**Maternal and Paternal Demographics**

*Maternal Education* **0.02** 0.76

Less than high school 0.62± 1.08 -0.008± 1.14

High school or more 0.21± 1.06 -0.06± 1.04

*Marital Status* 0.23 0.43

Married/Living with partner

0.24± 1.06 -0.07± 1.05

Single/Other 0.50± -1.09 0.09± 1.08

*Maternal Latinx Ethnicity*

Mexican/Central American (CA)

0.35± 1.08 0.14 -0.07± 0.95 0.86

Not Mexican/(CA) 0.17± -1.04 -0.05± 1.13

Caribbean/South American (SA)

/Spain-Portugal

0.23± 0.99 0.83 -0.07± 1.05 0.44

No Caribbean/SA/

Spain-Portugal 0.27± 1.08 0.05± 1.02

*Paternal Latinx Ethnicit*y

Mexican /CA 0.37± 1.16 0.18 -0.11± 0.97 0.50

Not Mexican /CA 0.21± 1.00 -0.03±-0.96

*Paternal Latinx Ethnicity*

SA/Caribbean

/Spain-Portugal 0.10± 1.07 0.41 -0.17± 1.00 0.58

No SA/Caribbean/

Spain-Portugal 0.28± 1.07 -0.05± 1.05

*Maternal Race/Ethnicity*

White 0.19± 1.11 0.60 -0.10± 0.91 0.92

Asian/Pacific Islander 0.17± 1.03 -0.02± 1.13

Black/Africa-American 0.73± 0.83 0.25±-0.77

Latinx 0.30± 1.06 -0.05± 1.10

*Any Parent – Mexican /Central American*

No 0.14± 1.02 **0.06** -0.08± 0.95 0.64

Yes 0.37± 1.09 -0.03± 1.13

*Maternal Age, years.* r=-0.06, p=0.26   r=0.003, p=0.96

*Maternal Age, years*  0.60 0.45

<20 0.51 ± 0.83 -0.63± 1.17

>=20 - <30 0.38± 1.14 -0.03± 1.20

>=30- <35 0.24± 1.04 -0.07± 0.94

>=35 0.20±1.06 -0.02± 1.04

*Age of Menarche* r=0.04, p=0.47 r=-0.03, p=0.58

<12 0.18± 1.15 0.59 0.03± 1.08 0.54

12 0.23± 0.99 -0.16± 0.91

>12 0.32± 1.05 -0.06± 1.08

**Maternal Health History**

*Maternal Pre-existing Diabetes Mellitus or Gestational*

Any diabetes 0.26± 1.05 0.94 0.13± 1.14 **0.09**

No diabetes 0.27± 1.11 -0.11± 1.02

*Maternal Pre-existing Hypertension or Gestational*

Any hypertension 0.75± 1.02 **<0.01** -0.0007± 1.09 0.67

No hypertension 0.16± 1.05 -0.07± 1.04

*Smoking*/*Secondhand Smoke*

Any smoke exposure 0.19± 1.03 0.71 -0.21± 1.13 0.38

No smoke exposure 0.27± 1.07 -0.04±-1.04

*Sugar Sweetened Beverages (SSB) Consumption, cups/wk*

r=0.09, **p=0.09**  r=0.05, p=0.34

*SSB Consumption, cups/wk*

0-<1 SSB cups per wk 0.14± 1.06 **0.0497** -0.10± 1.06 0.44

SSB >=1/ cups per wk 0.37± 1.06 -0.013± 1.04

*100% Fruit Juice, cups/wk*

r=0.06, p=0.25 r=-0.01, p=0.79

100% Fruit Juice, cups/wk

0 cups/wk 0.16± 1.00 0.21 0.02± 0.91 0.35

>0/wk 0.31± 1.09 -0.09± 1.11

*Mental Illness in Pregnancy*

No 0.27± 1.07 0.85 -0.01± 1.05 **0.06**

Yes 0.23± 1.06 - 0.32± 1.02

*Parity (continuous)* r=0.04, p=0.52 r=-0.04, p=0.51

Parity (categorical)

1 0.29+/-0.99 0.42 -0.005+/-0.98 0.61

2 0.16+/-1.15 -0.06+/-1.10

>=3 0.36+/-1.10 -0.16+/-1.13

*Medications for Depression*

*/Anxiety*

Yes 0.37+/-1.13 0.56 -0.14+/-1.04 0.64

No 0.25+/-1.05 -0.04+/-1.10

*Pre-Pregnancy Body Mass Index (BMI)*

r=0.04, p=0.47 r=0.10, **p=0.07**

*Pre-Pregnancy BMI (kg/m^2^)* Category

Underweight (<18) 0.90+/-1.22 **0.03** 0.36+/-1.19 **0.04**

Normal (>=18, <25) 0.24+/-0.99 -0.17+/-1.05

Overweight

(>=25 - <30) 0.03+/-1.17 -0.19+/-1.12

Obese (>= 30) 0.49+/-1.09 0.21+/-0.94

*Maternal Gestational Weight Gain, lbs*

r=-0.19, **p<0.01** r=-0006, p=0.91

*Maternal Weight Gain*

>40 lbs 0.11+/-1.02 0.37 0.04+/-1.06 0.44

<=40 lbs 0.27+/-1.06 -0.09+/-1.03

*American College of Obstetrics and Gynecology (ACOG) Gestational Weight Gain*

Below 0.28+/-1.04 0.16 -0.01+/-1.01 0.13

Within 0.10+/-1.08 -0.02+/-0.96

Over 0.41+/-1.05 -0.29+/-1.21

**Child Specifics**

*Gestational Age, wks*   r=-0.35, **p<0.01** r=0.22, **p<0.01**

*Gestational Age*

32-<35 wks 1.43+/-0.93 **<0.01** -0.74+/-1.32 <0.01

≥35 wks and <37 wks 0.74+/-0.78 0.71+/-0.89

≥37 wks 0.16+/-1.05 0.04+/-1.02

*Child Sex*

Female 0.34+/-1.06 0.19 0.06+/-1.05 **0.06**

Male 0.19+/-1.07 -0.16+/-1.04

*Apgar (5 mins*) r=-0.06, p=0.31 r=0.08, p=0.14

*Apgar (5 mins)*

<9 at 5 mins 0.24+/-1.06 0.39 -0.15+/-1.20 0.43

≥9 at 5 mins 0.24+/-1.06 -0.03+/-1.01

*Birth Type*

C-Section 0.16+/-1.18 0.36 -0.28+/-1.21 **0.03**

Vaginal 0.29+/-1.03 0.01+/-0.99

*Child Birthweight, Z* r=-0.48, **p<0.01** r=0.46, **p<0.01**

*Child Birthweight, kg* r=-0.48, **p<0.01** r=0.45**, p<0.01**

*Low Birthweight (<2500 g)*

Yes 1.21+/-0.81 **<0.01** -1.01+/-1.00 **<0.01**

No 0.19+/-1.05 0.02+/-1.02

*Macrosomic (>4000 g)*   **p<0.01**

Yes -0.99+/-0.79 0.68+/-0.85 **<0.01**

No 0.35+/-1.03 -0.10+/-1.04

*Leukocyte Telomere Length, T/S ratio*

r=0.12**, p=0.04** r=0.07, p=0.21

*Breastfeeding at Discharge*

Mixed feeding 0.51+/-1.14 **0.02** -0.17+/-1.05 0.25

Exclusive breastmilk 0.19+/-1.04 -0.007+/-1.03

*Breastfed at 4-6 weeks*  0.81

Yes 0.25+/-1.04 0.88 -0.07+/-1.03

No 0.28+/-1.23 -0.02+/-1.12

*Exclusively Breastfed, 4-6 weeks*

Yes 0.20 +/-1.08 0.28 0.06+/-1.03 **0.01**

No 0.33+/-1.02 -0.25+/-1.03
